# Risk of preterm birth and peripartal complications after first trimester termination of pregnancy: a retrospective cohort study of 35,897 singleton births

**DOI:** 10.1007/s00404-025-08242-w

**Published:** 2026-01-20

**Authors:** Caroline Helena Gabrysch, Livia Schirru, Wolfgang Henrich, Silke Wegener

**Affiliations:** Department of Obstetrics, Charité Universitätsmedizin Berlin Corporate Member of Freie Universität Berlin, Humboldt-Universität Zu Berlin, and Berlin Institute of Health (BIH), Berlin, Germany

**Keywords:** Termination of pregnancy, Preterm birth, Reproductive counseling, Peripartal hemorraghe

## Abstract

**Purpose:**

The objective of this study was to explore whether a history of termination of pregnancy (TOP) in births after 2015 in a high income setting is still linked to preterm birth (PTB) and peripartal complications.

**Methods:**

35,897 singleton births from a perinatal center with approximately 5000 births per year between 2015 and 2022 were analyzed. Patients with a history of first trimester TOP (TOP < 15 weeks) were compared to those who had never had a TOP. A two-step statistical approach using Chi-squared analysis and forward-step multiple logistic regression was used to explore the relationship.

**Results:**

4132 individuals (11.51%) had a history of first trimester TOP. Our findings suggest an association between past TOP and a higher risk for PTB (OR = 1.44, 95% CI [1.25–1.67], *p* < 0.001). This increases with the number of TOP, six or more TOP were associated with the highest odds ratio for spontaneous PTB (OR = 5.21, 95% CI [1.88–14.46], *p* = 0.002). The risk for PTB did not differ between methods. Furthermore, our data suggest an association between past TOP and placental retention (OR = 1.25, 95% CI [1.03–1.52],*p* = 0.022).

**Conclusion:**

These findings underscore the importance of still recognizing prior TOP as a risk factor in obstetric care. The results may inform targeted counseling and the development of preventative strategies to mitigate maternal and fetal morbidity.

**Supplementary Information:**

The online version contains supplementary material available at 10.1007/s00404-025-08242-w.

## What does this study add to the clinical work

**Table:** 

This study covers a gap in research that has not been investigated systematically in Germany since 2009—even though changes in TOP practice habits have taken place. Results indicate that TOP still increases the risk of PTB and peripartal complications in subsequent pregnancies.	

## Introduction

Termination of pregnancy (TOP) is a common gynecological procedure among women of reproductive age [1]. Possible long-term effects are uncertain, especially in relation to the risks for future pregnancies. In Germany, the rate of preterm births (PTB) is approximately 8%; in addition many women require hospitalization or treatment to prolong the pregnancy due to the risk of PTB [2]. Approximately 100,000 TOP (including vacuum aspiration (VA), dilation and curettage (D&C), and medical abortion (MA)) are performed every year in Germany, of which about 96% are in the first-trimester [3]. Globally, there has been an increased use of MA in recent years [4]. In Germany in 2022, MA made up 35.1% of all TOP, in contrast to 3.1% in 2000 [3, 5].

Furthermore, the increasing recognition of the risks associated with D&C, including damage to the endometrium or myometrium, has led to its decreased use as a primary method for TOP globally.

PTB, defined as delivery before 37 weeks of gestation, remains a major cause of infant morbidity and mortality worldwide, accounting for 18% of deaths in children under the age of five in 2019 (ages 2–4). Risk factors include smoking, low socioeconomic status, low BMI, maternal age below 18 years or older than 35 years, short cervical length, infertility treatments, and obstetric complications such as preeclampsia and cervical dilation associated with miscarriage or TOP [2, 5, 6].

Research on TOP and PTB has produced mixed results. Many studies have demonstrated a correlation between prematurity and previous induced or spontaneous abortion while several find none. Some studies have found a link between TOP and an increased risk of prematurity [6–11], while others report no correlation [12–16]. D&C has been associated with complications, and systematic reviews have identified it as a significant risk factor for PTB [17–19]. In contrast, studies focusing on medical abortion suggest that it may carry fewer risks for future pregnancies [20–22]. Other studies report no differences between methods [23]. Adverse outcomes such as low birthweight (LBW) and small for gestational age (SGA) have been associated with D&C and multiple terminations [14, 24]. In addition, data on increased risks with short intervals between TOP and next pregnancy has been described [25].

Current studies yield conflicting results regarding preterm premature rupture of membranes (PPROM). Holmlund et al. found no increased risk of PPROM following TOP [26]. Comparatively, Ancel et al. conducted the EUROPOP survey of 17 European countries and found that women with a history of TOP had an increased risk of PTB following PPROM [27].

Placental disorders, including placenta previa and accreta, linked to D&C or multiple TOP, may contribute to complications such as bleeding, PTB, and placental retention [28, 29]. However, conflicting findings exist, suggesting weak associations, possibly influenced by confounding factors [30].

Our study aims to investigate the clinical implications, exploring PTB risk, the need for preventative interventions as well as peri- and postpartum complications. Provision of these data, contributes to understanding of the long-term obstetric consequences of TOP in Germany.

## Methods

### Sample

35,897 singleton births that took place between 2015 and 2022 at a perinatal center were included in the study and were analyzed in a retrospective cohort analysis. Peripartal outcomes of patients with a history of first-trimester TOP (TOP < 15 weeks) were compared to those who had never had a TOP (control group). Inclusion criteria were age > 18 years at delivery. Exclusion criteria were a history of documented second or third trimester abortions. Furthermore, during the period of the analysis, several people gave birth multiple times. We inferred that including multiple births in the study would produce collinear and heteroscedastic results, therefore only the first birth of each woman within the timeframe was included in the analysis. Application of exclusion criteria is illustrated in Fig. 1S in the supplement. The study was approved by the Charité ethics committee (application number EA1/151/23), following the Helsinki declaration.

### Instruments

Data were extracted from clinical and sonographic documentation software Viewpoint 5 and 6 (GE HealthCare, Boston). Age, gestational history, pregnancy risks and pre-existing conditions, previous TOP, previous miscarriages, and previous uterine surgeries were retrieved. In case of multiparity, data on the previous births were obtained. The details on previous miscarriages and TOP were looked at when available, including the method used (curettage, vacuum aspiration or medical), gestational age at the time of the procedure, and the year in which the procedure was performed.

Weight gain, BMI as well as data on the index birth including preterm delivery, reason for preterm delivery, mode of delivery, fetal position, obstetric interventions, peripartal complications (bleeding, placental retention) were retrieved. Furthermore, data on attempts to prevent premature birth (tocolysis, cervical cerclage, and antenatal steroid prophylaxis) were assessed. Neonatal outcome data were gathered, including the APGAR-scores, size and gestational age at birth, intensive care treatment, laboratory results, and details on fetal lung maturation with antenatal steroids (ANS).

### Analyses

Demographic and medical characteristics were categorized and calculated as mean with standard deviation (SD). Chi-squared tests were administered to identify variables associated with PTB. For categories with 30 patients or less, Fisher’s exact test was performed. For analyses with larger sample sizes, Pearson’s Chi-squared test was conducted. For both types of test the exact 2-sided significance was considered. Subsequently, to discern the individual effects of various predictors, and to control for confounding variables, a multiple logistic regression (MLR) with a forward selection approach (fstep) analysis to explore the influence of multiple predictor variables on the single binary outcome of PTB was conducted. The predictor variables included maternal age, BMI, weight gain, gravida, para, as well as TOP and miscarriage history. Proportion of missing data for each variable is documented in Table 1S of the supplement. Odds ratio (OR) with confidence interval (CI) of 95% are reported.

In secondary analyses, the risk for spontaneous PTB based on method of TOP and the relationship between TOP and preventative measures for PTB was explored using a Chi-squared analysis followed by forward stepwise multiple logistic regression.

*p* Values < 0.05 were considered statistically significant. All statistical data analyses were conducted using SPSS (Version 27; IBM Corp., Armonk, NY, USA) and R (R Core Team, 2023) with RStudio (Posit PBC, Boston, MA, USA).

## Results

### Demographic characteristics and medical history

The demographic data of the cohort showed the mean age (31.46 years), mean BMI (24.43), mean number of previous pregnancies (gravida) (2.53), number of previous births (para) (1.97), and mean weight gain (13.61 kg) for the duration of the pregnancy. The descriptive statistics among the TOP and the control group were consistent, except for the mean gravida, which was notably higher in the TOP group, at 4.09 ± 2.10 pregnancies, compared to 2.39 ± 1.55 pregnancies for the control group.

### Primary analysis of previous TOP and preterm birth

**Fig. 1:**
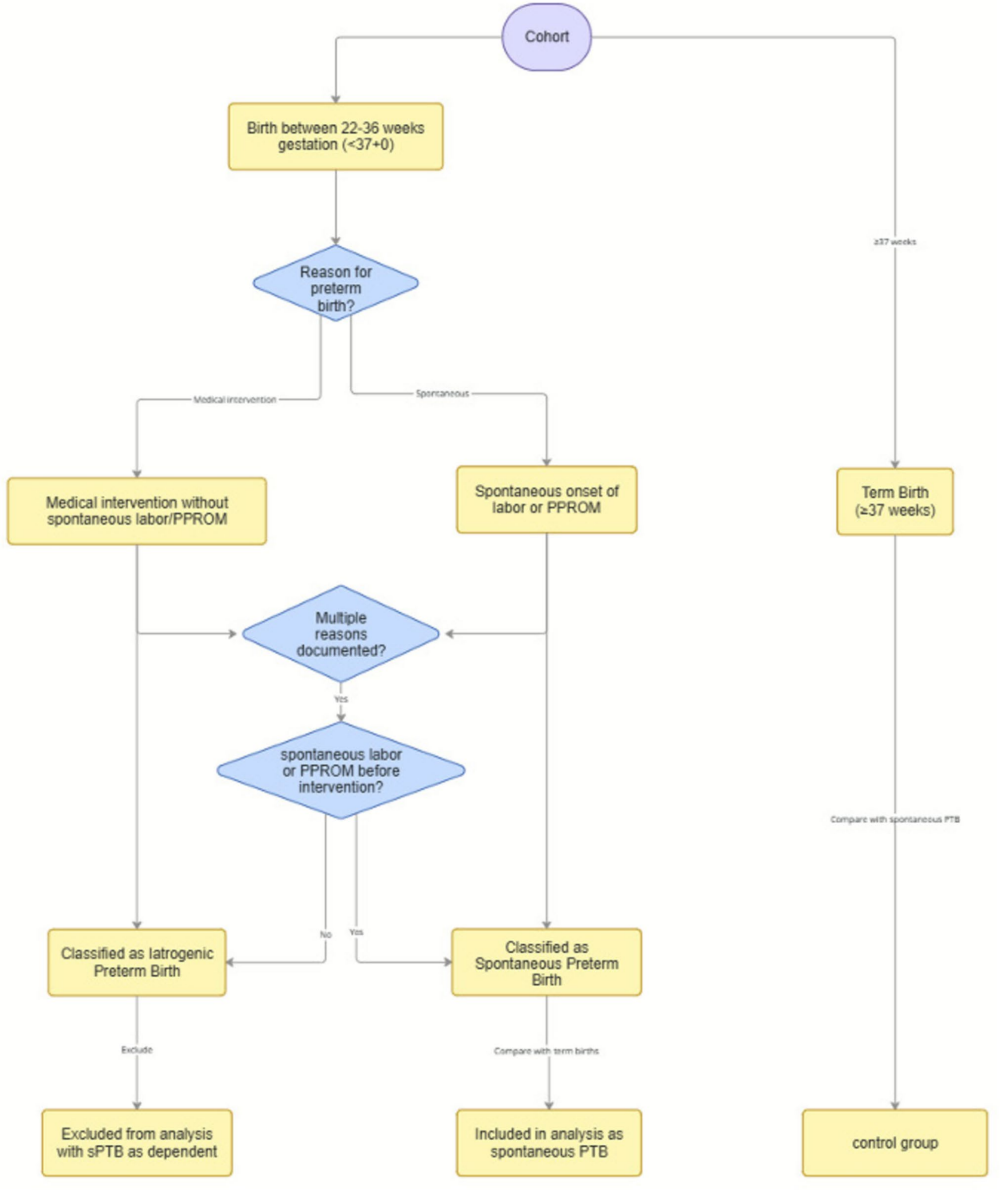
Definition criteria for the main analysis of PTB subgroups

When comparing the TOP and control groups, 7.8% of the TOP group had a spontaneous PTB, as opposed to 5.9% of the control group Table 1. For the analysis on PTB, the data were filtered for spontaneous PTB Iatrogenic PTBs were discounted from the analysis, this is illustrated in Fig. 1. After this, a sample size of 3903 individuals remained in the TOP group and 28,877 in the control group. Pearson’s Chi-squared analysis on previous TOP and spontaneous PTB, showed a significant result (*p* value < 0.001). Therefore, multiple logistic regression with spontaneous PTB as dependent variable was performed. Among other noise, previous TOP as a binary variable remained statistically significant (OR 1.44 (95% CI [1.25–1.67], *p* value < 0.001). Higher parity was found to have a statistically lower risk for PTB as compared to primiparity. Underweight individuals had a significantly higher risk for PTB, as well as those who gained little weight or lost weight during pregnancy (Table 2).

**Table 1:** Crosstabulation of number and proportion (%) of previous TOP (yes or no) and spontaneous preterm birth (yes or no)

Previous TOP	Term birth		Spontaneous PTB		Total	
	%	*n*	%	*n*	%	*n*
No	94.13	27,182	5.87	1695	100.00	28,877
Yes	92.19	3598	7.81	305	100.00	3903

*TOP* induced termination of pregnancy; *PTB* preterm birth; *n* sample size

**Table 2:** Multiple logistic regression for spontaneous preterm birth with TOP as a binary value

Variables		OR with 95% CI (lower–upper)*	*p* value
Parity	–	Total**	< 0.001
Primipara (0–1)	–	–	Reference
Moderate parity (2–3)	–	0.66 (0.59–0.74)	< 0.001
High parity (≥ 4)	–	0.65 (0.54–0.78)	< 0.001
BMI	–	Total**	< 0.001
Normal BMI	18.5 < x < 25 kg/m^2^	–	Reference
Underweight	< 18.5 kg/m^2^	1.22 (0.99–1.51)	n.s
Overweight	25 ≤ x < 30 kg/m^2^	0.85 (0.74–0.97)	0.017
Obese	30 ≤ x < 35 kg/m^2^	0.78 (0.647–0.96)	0.009
Morbidly obese	≥ 35 kg/m^2^	0.78 (0.61–0.99)	0.040
Previous miscarriage	–	1.19 (1.06–1.33)	0.004
Previous TOP	–	1.44 (1.25–1.67)	< 0.001
Weight gain***	–	Total**	< 0.001
Normal weight gain	10 ≤ x < 16 kg	–	Reference
Weight loss	≤ 0 kg	2.20 (1.40–3.44)	< 0.001
Little weight gain	0 < x < 10 kg	2.52 (2.23–2.33)	< 0.001
Moderate weight gain	16 < x < 20 kg	0.53 (0.44–0.64)	< 0.001
Significant weight gain	≥ 20 kg	0.39 (0.31–0.49)	< 0.001

^*^ Odds ratio (*OR*) with 95% confidence interval (CI) (lower–upper); **Total *p* value for the categorical variable; ***Weight gain during pregnancy, *n.s.*   not significant

In additional analyses the combined effect of prior miscarriages and TOPs showed a slightly higher impact on sPTB than TOP alone, results are demonstrated in Table S2 of the supplement.

### Number of TOP and PTB

**Fig. 2:**
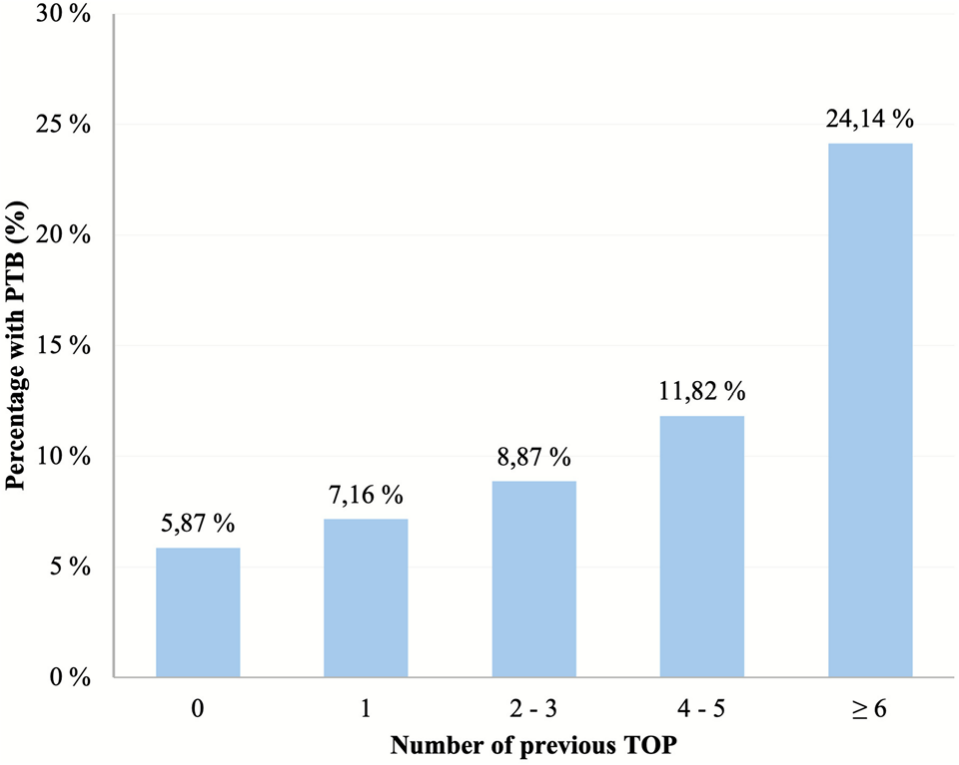
Number of induced terminations of pregnancy (TOPs) and incidence of spontaneous preterm birth (PTB). *TOP(s)* induced termination of pregnancy(s); *PTB* preterm birth

Within the TOP group 72.8% had undergone one TOP, 23.6% two to three TOP, 2.9% four to five TOP, and 0.8% had six or more TOP prior to the index pregnancy. This is illustrated in Fig. 2. To deepen the analysis, the relationship between the number of previous TOP and spontaneous PTB was explored. Crosstabulation of number of previous TOP and spontaneous PTBs was performed which showed that the proportion of spontaneous PTB increased with the number of abortions. This was analyzed with Pearson’s Chi-squared tests and was found to be statistically significant (*p* value < 0.001). Logistic regression considering clinical parameters was performed and showed a rising OR for PTB with the highest OR at six or more TOP (OR = 5.21, 95% CI [1.88–14.46, *p* = 0.002).

### Method of TOP and PTB

Pearson’s Chi-squared test was used to explore the relationship between the method of TOP (surgical (curettage and vacuum aspiration) and medical (mifepristone with misoprostol)), as well as those where the method of abortion was not documented. This analysis showed a higher proportion of spontaneous PTB across all methods, but no statistical significance was found.

### Previous TOP and intervention for PTB

Risk of interventions to prevent PTB in the TOP and the control group was explored. While statistical analysis suggested potential associations of ANS, oral tocolysis and cerclage with TOP, no robust association was identified.

### Previous TOP and placental disorders

Chi-squared test indicated an increased risk of placental retention (*p* value = 0.005) and placenta previa (*p* value = 0.013) among the TOP group when compared to the control group; this is demonstrated in Table 3.

**Table 3:** Chi-square test on previous TOP and placental disorders

Variables	Prior TOP		No prior TOP		*p* value
	%	*n*	%	*n*	
Placental retention	3.93	157 (*N* = 3996)	3.08	894 (*N* = 29,037)	**0.005
Placental abruption	0.34	14 (*N* = 4132)	0.19	57 (*N* = 30,192)	**n.s
Placental insufficiency	0.05	2 (*N* = 4132)	0.06	19 (*N* = 30,192)	*n.s
Placenta praevia	0.44	18 (*N* = 4132)	0.22	67 (*N* = 30,192)	**0.013
Placenta accreta spectrum	0.13	2 (*N* = 1490)	0.28	29 (*N* = 10,524)	**n.s

*TOP* termination of pregnancy; *n* sample size, * Fisher’s exact test; **Chi-square test, *n.s.* non significant. Percentages are shown as % and *n*/*N*, where n is the number of individuals with the variable, and N is the total number of individuals eligible within each group. The total number included (*N*) varies between variables due to missing data

The logistic regression analysis for placental retention showed that a previous TOP was significantly associated (OR 1.25 (95% CI [1.03–1.52], *p* value = 0.022). Furthermore, age, (moderate) parity, and a history of miscarriage or previous cesarean section were significantly associated with placental retention. Placenta previa was no longer significantly associated with TOP in logistic regression.

### Previous TOP and peripartum bleeding

Overall, previous termination of pregnancy (TOP) was not associated with a significant increase in peripartum blood loss—except in those cesarean deliveries complicated by specific placental pathologies, where prior TOP further amplified the risk (See Table 4). The threshold for increased peripartal bleeding was set at ≥ 500 mL for vaginal deliveries and ≥ 1000 mL for caearean births. Multiple regression for cesarean sections showed a significantly elevated OR for blood loss in deliveries with placental abruption (OR 11.24 (95% CI [4.28–29.51], *p* value < 0.001), placental retention (OR 11.58 (95% CI [3.83–35.03], *p* value < 0.001) and placenta pravia (OR 22.45 (95% CI [3.09–163], *p* value = 0.002). Results are demonstrated in Table 3S of the supplementary material.

**Table 4:** Chi-square tests for the association between previous TOP and hemorrhage, uterine rupture, and imminent uterine rupture

Variables	Prior TOP		No prior TOP		*p* value
	%	*n*	%	*n*	
Blood loss ≥ 500 mL (vaginal deliveries)	12.01	288 (*N* = 2398)	11.98	2295 (*N* = 19,157)	**n.s
Blood loss ≥ 1000 mL (caesarean births)	5.91	96 (*N* = 1624)	4.70	482 (*N* = 10,253)	**0.040
Uterine rupture	0.02	1 (*N* = 4131)	0.02	6 (*N* = 30,192)	*n.s
Imminent uterine rupture	0.05	2 (*N* = 4130)	0.02	7 (*N* = 30,192)	*n.s

*TOP* termination of pregnancy; *Fisher’s exact test; **Chi-square; Percentages are shown as % and *n*/*N*, where n is the number of individuals with the complication, and *N* is the total number of individuals eligible within each group. The total number included (*N*) varies between variables due to missing data

## Discussion

### Main findings

4,132 individuals (11.51 %) had a history of first trimester TOP. Results indicated that a past TOP increased the risk for spontaneous PTB in future pregnancy (OR = 1.44, 95 % CI [1.25–1.67], *p* < 0.001). This association increased with the number of TOP, with six or more past TOP being most strongly associated with an increased risk of PTB (OR = 5.21, 95 % CI [1.88–14.46], *p* = 0.002). The risk for PTB did not differ between TOP methods. Furthermore, our data indicate that past TOP elevates the risk for placental retention (OR = 1.25, 95 % CI [1.03–1.52], *p* = 0.022). Of the other peripartum complications examined, none were significantly linked to previous TOP.

### Strengths and limitations

This study demonstrates several notable strengths. First, the large sample size of over 35,000 singleton births enhances statistical power and contributes to the robustness of the findings. Second, the use of multiple logistic regression allowed for detailed analysis of predictor variables while controlling for important confounders such as maternal age, BMI, parity, and gestational weight gain. Third, the study focused on clinically relevant outcomes: in addition to assessing the risk of PTB, it also examined associated complications, including placental retention and the need for tocolysis. Lastly, the analysis is based on contemporary data from 2015 to 2022, reflecting current clinical practices and advancements in obstetric care.

Despite these strengths, several limitations should be considered. Within a retrospective observational study, causal relationships cannot be established. Data quality depends on the completeness and accuracy of existing clinical documentation. While many important variables were controlled for, residual confounding remains possible. Possible confounders such as educational or migration background, maternal chronic conditions or smoking status were not or only sporadically documented in the database that derived from an ultrasound documentation program and therefore were not included in the regression model.

All women had health insurance coverage; however, socioeconomic status could not be assessed in sufficient detail due to incomplete and inconsistently documented retrospective data. Across the entire cohort, only 41 women in the PTB group reported a history of conization. We therefore did not include prior conization as a covariate in our analyses. This decision was driven by substantial missing clinical detail: the operative technique (e.g., cold-knife conization vs. LEEP/LLETZ), extent of excision (depth/volume), number of procedures, and interval to pregnancy were not available. Since PTB risk appears to differ by technique and is strongly influenced by excision depth/volume, treating “conization” as a single binary exposure would likely introduce non-differential misclassification and yield results that are difficult to interpret. In addition, detailed information on the underlying cervical disease (e.g., grade of dysplasia/CIN, indication, margins) was not sufficiently captured, raising concern for confounding by indication and limiting meaningful adjustment. Their absence might introduce residual confounding, and further studies, including a more complete data set, are warranted. Furthermore, although the overall sample size was large, the study may still be underpowered to detect significant associations with rare complications, limiting the interpretability of findings in those subgroups.

### Comparison with the literature

Our research highlights that patients with a history of first trimester TOP had an increased risk of spontaneous PTB in subsequent pregnancies. This finding is in line with the literature, that highlights the elevated risk especially for pregnancies following D&C [7, 17, 19]. As stated, the available retrospective data were not available on every possible factor contributing to PTB, so interpregnancy interval, prior unsafe abortions or underreporting might have influenced the outcome of our sample [25, 31].

In our sample, this risk rose with the number of previous TOP, though it did not differ between methods of TOP. Two studies from Finland found similar risk-profiles for both medical and surgical TOP [14, 23], while Saccone et al. found that medical TOP results in the same risk profile as primigravidas while surgical TOP leads to an increased risk of PTB [17]. While no difference between medical and surgical procedures was found in a study by Kc et al., an elevated risk for PTB after later abortions (second trimester or higher) was identified [32]. In a systematic review Gan et al. interestingly describe no difference in the risk of PTB between the methods of TOP, but an increased risk for miscarriage and postpartum hemorrhage in the group of surgical TOP [20]. Another factor that must be considered is that the methods of TOP have been evolving over the last decades. Not only a shift toward more medical abortions, that will result in long-term data, has been described [21], but it also might be misleading to compare risks of surgical procedures performed 20 years apart. A Scottish study found increased rates of PTB in a cohort from 1980 to 1990, a result that could not be reproduced in 2008, suggesting that modernized methods of TOP contribute to the decreased rates of PTB [22]. The same question must be kept in mind when addressing medical TOP, on which German data from 1998 to 2000 suggest an elevated risk for PTB after medical TOP [24]. We aimed to analyze whether the need for medical intervention such as tocolysis, cerclage and steroids to prevent or prepare for PTB was elevated in women with a history of TOP, even if the procedure had been successful and PTB could be prevented. Although our data suggest an increased level of interventions in pregnancies with a history of TOP, limitations in our data set do not allow to draw clinical conclusions. Given the extent of these interventions and their impact on maternal stress and hospitalization, including associated side effects and costs, further research on this aspect is warranted.

In our sample, an increased risk of placental retention was found in the TOP group, but no other peripartal complications were significantly associated with prior TOP. Our observation of placental retention is in line with data published by Zhou et al. [28]. No association between TOP and placental abruption was observed. Data on a possible association of placental abruption and TOP are inconclusive. Several studies report an association [18, 29], while others did not find a significant link [33].

An increased risk of placenta previa in women with a history of TOP has been described [33, 34]. After correcting for other influential variables our data did not show a significant correlation between a history of TOP and subsequent placenta previa.

While a monocausal explanation for preterm birth and placental retention is unlikely, several studies suggest potential biological mechanisms that support our findings. Any kind of cervical trauma has been described as a risk factor for preterm birth [35]. Apart from mechanical pathways, data suggest that uterine procedures can promote a proinflammatory intrauterine environment [36], that might lead to complications and is also linked to abnormal placentation [37]. The slightly increased effect on sPTB that our data show for the combined effect of previous miscarriages and TOPs points toward underlying mechanical and biochemical effects of intrauterine procedures that might prevail.

Our data did not indicate a direct association between previous TOP and increased peripartum blood loss in either vaginal or cesarean deliveries. However, given the observed association between prior TOP and placental retention—a known risk factor for increased intrapartum and postpartum bleeding—an elevated risk of peripartum hemorrhage in women with a history of TOP should be considered.

### Clinical implications

Key clinical implications emphasize the necessity for adequate patient counseling and informed consent. It is crucial that healthcare providers offer comprehensive information on the potential risks associated with TOP, especially in women with prior PTB. The study underscores the importance of thorough documentation and careful consideration in patient management, given the stigmatized nature of the topic and the potential for underreporting previous TOP procedures.

## Conclusion

Preterm birth remains a significant etiology of neonatal morbidity and mortality, and induced pregnancy termination in the first trimester is one of the most frequent gynecological procedures among reproductive-aged people. Familiarization with potential late obstetric complications is therefore necessary to enable optimization of patient care and adequate informed consent. This study tries to cover a gap in research that has not been investigated systematically in Germany since 2009—even though changes in TOP practice habits have taken place. Yet, the range of the research can be enhanced through inclusion of wider recognitions of key variables such as the method of TOP used, specific gestational age at the time of the procedure and complication or follow-up information. Having a wider recognition of such factors would improve the ability to predict complications. Improvement and standardization of both clinical and preclinical documentation should be prioritized. Future studies compromising rural areas with probable worse coverage, including exact documentation of medical abortion and subtype of surgical abortion using contemporary techniques are long overdue. A planned strategic approach to obtaining and analyzing this data must be established, to prevent underreporting TOP in obstetric records due to ongoing stigmatization.

Terminations of pregnancy are medically safe procedures with a low risk of complications, particularly with modern methods; however, reduction of unintended pregnancies by making contraception widely available and introduction of targeted counseling for women with a history of repeated TOP is essential.

## Supplementary Information

Below is the link to the electronic supplementary material.

Supplementary file1 (DOCX 243 KB)

## Data Availability

All data generated or analyzed during this study are included in this article. Further enquiries can be directed to the corresponding author.

## References

[1] Sedgh G, Bearak J, Singh S, Bankole A, Popinchalk A, Ganatra B, Rossier C, Gerdts C, Tunçalp Ö, Johnson BR Jr., Johnston HB, Alkema L (2016) Abortion incidence between 1990 and 2014: global, regional, and subregional levels and trends. Lancet 388(10041):258–267. 10.1016/s0140-6736(16)30380-4 27179755 10.1016/S0140-6736(16)30380-4 PMC5498988

[2] Bundesauswertung zum Erfassungsjahr 2020 Geburtshilfe Qualitätsindikatoren und Kennzahlen. IQTIG—Institut für Qualitätssicherung und Transparenz im Gesundheitswesen; 2021.

[3] Fachserie Schwangerschaftsabbrüche. Germany: Statistisches Bundesamt; 2022 26/01/2023. Contract No.: 2120300217004. Available from: https://www.destatis.de/DE/Themen/Gesellschaft-Umwelt/Gesundheit/Schwangerschaftsabbrueche/_inhalt.html#_3wp4jklfz; 26/01/2023 accessed:

[4] Popinchalk A, Sedgh G (2019) Trends in the method and gestational age of abortion in high-income countries. BMJ Sex Reprod Health 45(2):95–103. 10.1136/bmjsrh-2018-200149 30962177 10.1136/bmjsrh-2018-200149 PMC6579506

[5] Schwangerschaftsabbrüche 2000. Statistisches Bundesamt; 2007. Available from: https://www.statistischebibliothek.de/mir/receive/DEHeft_mods_00008540; accessed:

[6] Brown JS Jr, Adera T, Masho SW (2008) Previous abortion and the risk of low birth weight and preterm births. J Epidemiol Community Health 62(1):16–22. 10.1136/jech.2006.050369 18079328 10.1136/jech.2006.050369

[7] Shah PS, Zao J (2009) Induced termination of pregnancy and low birthweight and preterm birth: a systematic review and meta-analyses. BJOG 116(11):1425–1442. 10.1111/j.1471-0528.2009.02278.x 19769749 10.1111/j.1471-0528.2009.02278.x

[8] Hardy G, Benjamin A, Abenhaim HA (2013) Effect of induced abortions on early preterm births and adverse perinatal outcomes. J Obstet Gynaecol Can 35(2):138–143. 10.1016/s1701-2163(15)31018-5 23470063 10.1016/S1701-2163(15)31018-5

[9] Zafran N, Musa M, Zuarez-Easton S, Garmi G, Romano S, Salim R (2017) Risk of preterm birth and low birthweight following consecutive surgical and medical abortions. Arch Gynecol Obstet 296(4):763–769. 10.1007/s00404-017-4474-x 28756529 10.1007/s00404-017-4474-x

[10] Moreau C, Kaminski M, Ancel PY, Bouyer J, Escande B, Thiriez G, Boulot P, Fresson J, Arnaud C, Subtil D, Marpeau L, Rozé JC, Maillard F, Larroque B (2005) Previous induced abortions and the risk of very preterm delivery: results of the EPIPAGE study. BJOG 112(4):430–437. 10.1111/j.1471-0528.2004.00478.x 15777440 10.1111/j.1471-0528.2004.00478.x

[11] Voigt M, Olbertz D, Fusch C, Krafczyk D, Briese V, Schneider KT (2008) The influence of previous pregnancy terminations, miscarriages and still-births on the incidence of babies with low birth weight and premature births as well as a somatic classification of newborns. Z Geburtshilfe Neonatol 212(1):5–12. 10.1055/s-2008-1004690 18293256 10.1055/s-2008-1004690

[12] Parazzini F, Cipriani S, Chiaffarino F, Sandretti F, Bortolus R, Chiantera V (2007) Induced abortion and risk of small-for-gestational-age birth. BJOG 114(11):1414–1418. 10.1111/j.1471-0528.2007.01226.x 17803719 10.1111/j.1471-0528.2007.01226.x

[13] Parazzini F, Ricci E, Chiaffarino F, Cipriani S, Tozzi L, Fedele L (2010) Does induced abortion increase the risk of preterm birth? Results from a case-control study. Gynecol Obstet Invest 69(1):40–45. 10.1159/000253848 19887819 10.1159/000253848

[14] Kc S, Hemminki E, Gissler M, Virtanen SM, Klemetti R (2017) Perinatal outcomes after induced termination of pregnancy by methods: a nationwide register-based study of first births in Finland 1996-2013. PLoS ONE 12(9):e0184078. 10.1371/journal.pone.0184078 28863151 10.1371/journal.pone.0184078 PMC5593514

[15] Ke L, Lin W, Liu Y, Ou W, Lin Z (2018) Association of induced abortion with preterm birth risk in first-time mothers. Sci Rep 8(1):5353. 10.1038/s41598-018-23695-7 29599500 10.1038/s41598-018-23695-7 PMC5876335

[16] Woolner A, Bhattacharya S, Bhattacharya S (2014) The effect of method and gestational age at termination of pregnancy on future obstetric and perinatal outcomes: a register-based cohort study in Aberdeen, Scotland. BJOG 121(3):309–318. 10.1111/1471-0528.12455 24148689 10.1111/1471-0528.12455

[17] Saccone G, Perriera L, Berghella V (2016) Prior uterine evacuation of pregnancy as independent risk factor for preterm birth: a systematic review and metaanalysis. Am J Obstet Gynecol 214(5):572–591. 10.1016/j.ajog.2015.12.044 26743506 10.1016/j.ajog.2015.12.044

[18] McCarthy FP, Khashan AS, North RA, Rahma MB, Walker JJ, Baker PN, Dekker G, Poston L, McCowan LM, O’Donoghue K, Kenny LC (2013) Pregnancy loss managed by cervical dilatation and curettage increases the risk of spontaneous preterm birth. Hum Reprod 28(12):3197–3206. 10.1093/humrep/det332 24052504 10.1093/humrep/det332

[19] Lemmers M, Verschoor MA, Hooker AB, Opmeer BC, Limpens J, Huirne JA, Ankum WM, Mol BW (2016) Dilatation and curettage increases the risk of subsequent preterm birth: a systematic review and meta-analysis. Hum Reprod 31(1):34–45. 10.1093/humrep/dev274 26534897 10.1093/humrep/dev274

[20] Gan C, Zou Y, Wu S, Li Y, Liu Q (2008) The influence of medical abortion compared with surgical abortion on subsequent pregnancy outcome. Int J Gynaecol Obstet 101(3):231–238. 10.1016/j.ijgo.2007.12.009 18321519 10.1016/j.ijgo.2007.12.009

[21] Magro Malosso ER, Saccone G, Simonetti B, Squillante M, Berghella V (2018) US trends in abortion and preterm birth. J Matern Fetal Neonatal Med 31(18):2463–2467. 10.1080/14767058.2017.1344963 28629238 10.1080/14767058.2017.1344963

[22] Oliver-Williams C, Fleming M, Monteath K, Wood AM, Smith GC (2013) Changes in association between previous therapeutic abortion and preterm birth in Scotland, 1980 to 2008: a historical cohort study. PLoS Med 10(7):e1001481. 10.1371/journal.pmed.1001481 23874161 10.1371/journal.pmed.1001481 PMC3706322

[23] Männistö J, Mentula M, Bloigu A, Hemminki E, Gissler M, Heikinheimo O, Niinimäki M (2013) Medical versus surgical termination of pregnancy in primigravid women—is the next delivery differently at risk? A population-based register study. BJOG 120(3):331–337. 10.1111/1471-0528.12034 23126244 10.1111/1471-0528.12034

[24] Voigt M, Henrich W, Zygmunt M, Friese K, Straube S, Briese V (2009) Is induced abortion a risk factor in subsequent pregnancy? J Perinat Med 37(2):144–149. 10.1515/jpm.2009.001 18976047 10.1515/JPM.2009.001

[25] Männistö J, Bloigu A, Mentula M, Gissler M, Heikinheimo O, Niinimäki M (2017) Interpregnancy interval after termination of pregnancy and the risks of adverse outcomes in subsequent birth. Obstet Gynecol 129(2):347–354. 10.1097/aog.0000000000001836 28079768 10.1097/AOG.0000000000001836

[26] Holmlund S, Kauko T, Matomäki J, Tuominen M, Mäkinen J, Rautava P (2016) Induced abortion—impact on a subsequent pregnancy in first-time mothers: a registry-based study. BMC Pregnancy Childbirth 16(1):325. 10.1186/s12884-016-1109-3 27776483 10.1186/s12884-016-1109-3 PMC5078979

[27] Ancel PY, Lelong N, Papiernik E, Saurel-Cubizolles MJ, Kaminski M (2004) History of induced abortion as a risk factor for preterm birth in European countries: results of the EUROPOP survey. Hum Reprod 19(3):734–740. 10.1093/humrep/deh107 14998979 10.1093/humrep/deh107

[28] Zhou W, Nielsen GL, Larsen H, Olsen J (2001) Induced abortion and placenta complications in the subsequent pregnancy. Acta Obstet Gynecol Scand 80(12):1115–1120. 10.1034/j.1600-0412.2001.801207.x 11846708 10.1034/j.1600-0412.2001.801207.x

[29] Scholten BL, Page-Christiaens GC, Franx A, Hukkelhoven CW, Koster MP (2013) The influence of pregnancy termination on the outcome of subsequent pregnancies: a retrospective cohort study. BMJ Open. 10.1136/bmjopen-2013-002803 23793655 10.1136/bmjopen-2013-002803 PMC3669713

[30] Zhu QX, Gao ES, Chen AM, Luo L, Cheng YM, Yuan W (2009) Mifepristone-induced abortion and placental complications in subsequent pregnancy. Hum Reprod 24(2):315–319. 10.1093/humrep/den426 19054774 10.1093/humrep/den426

[31] Haddad LB, Nour NM (2009) Unsafe abortion: unnecessary maternal mortality. Rev Obstet Gynecol. 2(2):122–126 19609407 PMC2709326

[32] Kc S, Gissler M, Klemetti R (2020) The duration of gestation at previous induced abortion and its impacts on subsequent births: a nationwide registry-based study. Acta Obstet Gynecol Scand 99(5):651–659. 10.1111/aogs.13788 32128786 10.1111/aogs.13788

[33] Lowit A, Bhattacharya S, Bhattacharya S (2010) Obstetric performance following an induced abortion. Best Pract Res Clin Obstet Gynaecol 24(5):667–682. 10.1016/j.bpobgyn.2010.02.015 20362515 10.1016/j.bpobgyn.2010.02.015

[34] Jenabi E, Salimi Z, Bashirian S, Khazaei S, Ayubi E (2022) The risk factors associated with placenta previa: an umbrella review. Placenta 117:21–27. 10.1016/j.placenta.2021.10.009 34768164 10.1016/j.placenta.2021.10.009

[35] Vink J, Feltovich H (2016) Cervical etiology of spontaneous preterm birth. Semin Fetal Neonatal Med 21(2):106–112. 10.1016/j.siny.2015.12.009 26776146 10.1016/j.siny.2015.12.009 PMC4798922

[36] Couture C, Brien ME, Boufaied I, Duval C, Soglio DD, Enninga EAL, Cox B, Girard S (2023) Proinflammatory changes in the maternal circulation, maternal-fetal interface, and placental transcriptome in preterm birth. Am J Obstet Gynecol 228(3):332.e1-e17. 10.1016/j.ajog.2022.08.035 36027951 10.1016/j.ajog.2022.08.035

[37] Greenbaum S, Wainstock T, Dukler D, Leron E, Erez O (2017) Underlying mechanisms of retained placenta: evidence from a population based cohort study. Eur J Obstet Gynecol Reprod Biol 216:12–17. 10.1016/j.ejogrb.2017.06.035 28692888 10.1016/j.ejogrb.2017.06.035

