# Supplementary material for: Risk of preterm birth and peripartal complications after first trimester termination of pregnancy: a retrospective cohort study of 35,897 singleton births
Source: Arch Gynecol Obstet. 2026 Jan 20;313(1):55. doi: 10.1007/s00404-025-08242-w (PMC12819516; doi:10.1007/s00404-025-08242-w)
Supplement: Supplementary file 1 — Supplementary file1 (DOCX 243 KB) [file 404_2025_8242_MOESM1_ESM.docx]

# **Supplement**

# **Risk of Preterm Birth and Peripartal Complications After First Trimester Termination of Pregnancy: A Retrospective Cohort Study of 35,897 Singleton Births**

Caroline Gabrysch^1^, Livia Schirru^1^, Wolfgang Henrich^1^, Silke Wegener^1^

^1^Departement of Obstetrics, Charité Universitätsmedizin Berlin

***Table 1S*** *Missing data for variables included in the analysis*

| Variable | Missing data | |
| --- | --- | --- |
|  | n | % |
| TOP | 1573 | 4.4 |
| sPTB | 1622 | 4.5 |
| Parity | 2 | 0.006 |
| BMI | 928 | 2.6 |
| Weight | 5772 | 16.1 |
| Blood loss > 1000 (CS) | 431 | 3.4 |
| Blood loss > 500 (VB) | 480 | 2.1 |
| Placental abruption | 0 | 0 |
| Placental retention | 0 | 0 |
| Placenta praevia | 0 | 0 |

***Table 2S*** *Multiple logistic regression for spontaneous preterm birth with combined variable TOP and miscarriage as a binary value.*

| Variables | | | OR with 95 % CI  (lower-upper)* | *p*-value |
| --- | --- | --- | --- | --- |
| Parity | - | | Total** | < 0.001 |
| Primipara (0-1) | - | | - | Reference |
| Moderate parity (2-3) | - | | 0.67 (0.60-0.75) | < 0.001 |
| High parity (≥ 4) | - | | 0.67 (0.56-0.80) | < 0.001 |
| BMI | - | | Total** | < 0.001 |
| Normal BMI | 18.5 < x < 25 kg/m² | | - | Reference |
| Underweight | < 18.5 kg/m² | | 1.22 (0.99-1.51) | n.s. |
| Overweight | 25 ≤ x < 30 kg/m² | | 0.85 (0.74-0.98) | 0.021 |
| Obese | 30 ≤ x < 35 kg/m² | | 0.79 (0.65-0.95) | 0.012 |
| Morbidly obese | ≥ 35 kg/m² | | 0.79 (0.62-1.00) | 0.050 |
| Previous TOP*miscarriage | |  | 1,47 (1,13-1.91) | 0.004 |
| Weight gain*** | - | | Total** | < 0.001 |
| Normal weight gain | 10 ≤ x < 16 kg | | - | Reference |
| Weight loss | ≤ 0 kg | | 2.21 (1.41-3.45) | < 0.001 |
| Little weight gain | 0 < x < 10 kg | | 2.52 (2.24-2.84) | < 0.001 |
| Moderate weight gain | 16 < x < 20 kg | | 0.53 (0.44-0.64) | < 0.001 |
| Significant weight gain | ≥ 20 kg | | 0.40 (0.31-0.50) | < 0.001 |

* Odds ratio (*OR*) with 95% confidence interval (CI) (lower-upper); **Total *p*-value for the categorical variable; ***Weight gain during pregnancy, n.s. = not significant

***Table 3S*** *Multiple logistic regression for blood loss ≥ 1,000 mL (caesarean deliveries).*

| Variable | OR with 95 % CI  (lower-upper)* | *p*-value |
| --- | --- | --- |
| Placental abruption | 11.24 (4.28-29.51) | < 0.001 |
| Placental retention | 11.58 (3.83-35.03) | < 0.001 |
| Placenta praevia | 22.45 (3.09-163.00) | 0.002 |

* Odds ratio (*OR*) with 95 % confidence interval (CI) (lower-upper)


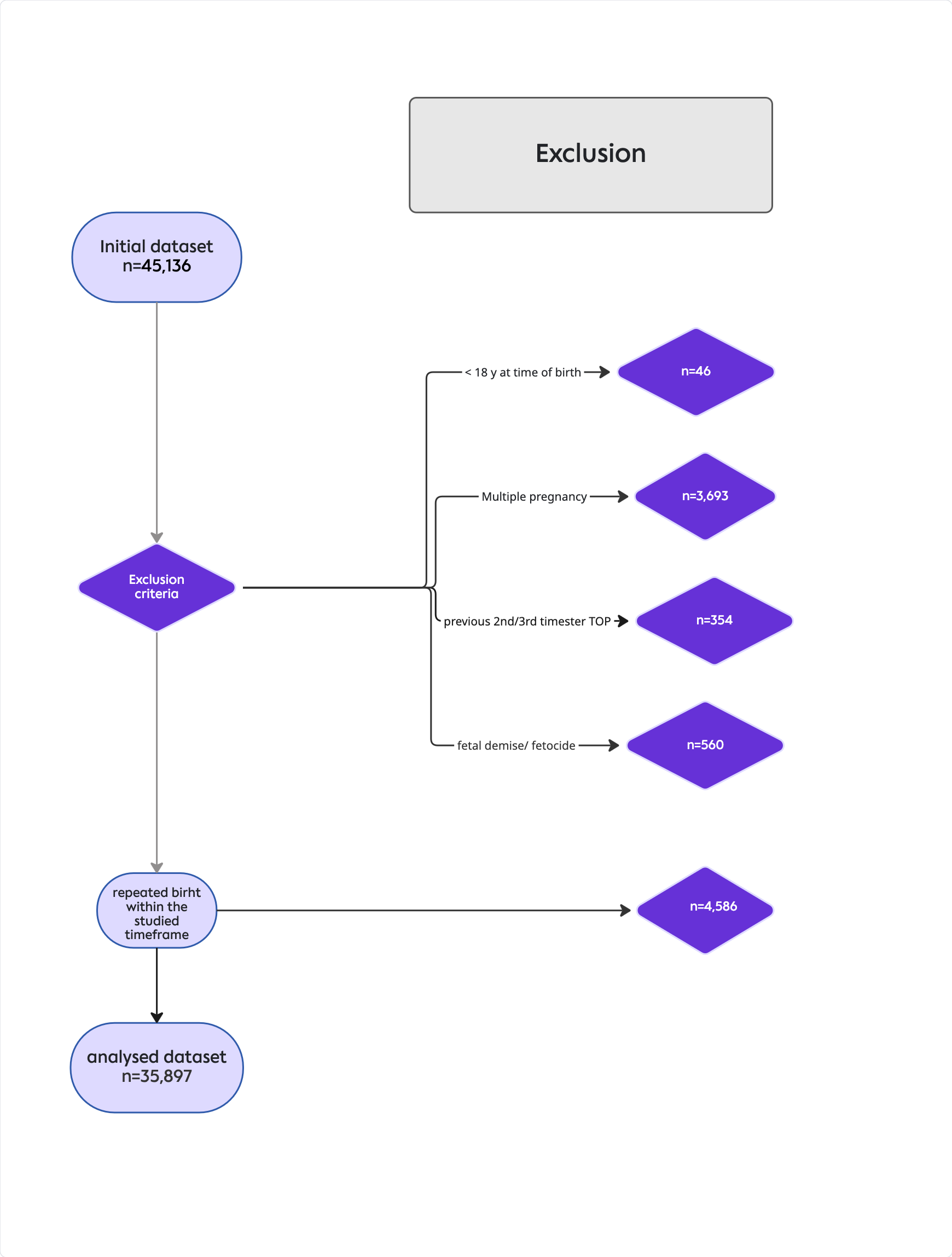


Figure 1S Flowchart illustrating the exclusion criteria applied to the dataset.
